# Pacing and Performance in the 6 World Marathon Majors

**DOI:** 10.3389/fspor.2019.00054

**Published:** 2019-11-08

**Authors:** José Joaquín Díaz, Andrew Renfree, Eduardo J. Fernández-Ozcorta, Miguel Torres, Jordan Santos-Concejero

**Affiliations:** ^1^Department of Physical Education and Sport, University of the Basque Country UPV/EHU, Vitoria-Gasteiz, Spain; ^2^School of Sport and Exercise Science, University of Worcester, Worcester, United Kingdom; ^3^Research Group HUM-643, Department of Integrated Didactics, University of Huelva, Huelva, Spain; ^4^Department of Energy Engineering, University of Seville, Seville, Spain

**Keywords:** long-distance running, endurance, athletics, tactics, pacing

## Abstract

The main goal of this study was to analyse the pacing strategies displayed by the winners of the six World Marathon Majors in order to determine which race offers the greatest potential for future world record attempts. For data analysis, the total distance of the marathon was divided into eight sections of 5 km and a final section of 2.195 km, and time needed to complete each section was calculated in seconds. When we analyzed the mean winning time in the last 13 editions of each of the World Marathon Majors, we observed differences between New York and London (ES = 1.46, moderate effect, *p* = 0.0030), New York and Berlin (ES = 0.95, small effect, *p* = 0.0001), London and Boston (ES = 0.08, small effect, *p* = 0.0001), Boston and Berlin (ES = 0.10, small effect, *p* = 0.0001), Boston and Chicago (ES = 0.16, small effect, *p* = 0.0361), Berlin and Tokyo (ES = 0.20, small effect, *p* = 0.0034), Berlin and Chicago (ES = 0.27, small effect, *p* = 0.0162). This study shows that Berlin and London are likely candidates for future world record attempts, whilst such a performance is unlikely in New York or Boston.

## Introduction

The pacing strategy adopted during competition is crucial in determining marathon running performance (Abbiss and Laursen, 2008; Ely et al., 2008; March et al., 2011; Renfree and Gibson, 2013). Pacing, which has been described as the ability to use and distribute energy resources efficiently during athletic competition (Foster et al., 1993), aims to optimize the use of physiological reserves before the end of the race. This would help in avoiding premature fatigue (Skorski and Abbiss, 2017) and a subsequent reduction in speed before task completion (Foster et al., 2004; Hettinga et al., 2007; Tucker and Noakes, 2009).

Different studies have identified a number of factors that influence pacing during a marathon race, including changes in terrain or altitude (Haney and Mercer, 2011), as well as environmental and body temperature (Marino et al., 2004; El Helou et al., 2012; Hoogkamer et al., 2017). To date, there is no consensus about the mechanisms through which the regulation of pace is achieved (Renfree et al., 2015), although factors such as the assessment of perceived exertion (Tucker and Noakes, 2009), the Hazard score (de Koning et al., 2011), and emotion (Baron et al., 2011; Venhorst et al., 2018) have all been studied.

Recently, it has been established that the pacing strategy adopted has a major influence on the performances achieved by world record breaking athletes (Díaz et al., 2018). In recent years, the world records have been achieved through progressively more consistent strategies than was the case in record performances of more than 25 years ago, which were typically characterized by a progressive reduction in speed (Díaz et al., 2018, 2019).

The present world record holder, Eliud Kipchoge of Kenya (2:01:39, Berlin Marathon, 16 September 2018) succeeded in lowering the previous record by 1 min and 18 s. This performance has increased speculation regarding the possibility of a sub 2 h performance (Hoogkamer et al., 2017; Sousa et al., 2018). Some authors and scientists have suggested that such a performance is physiologically impossible (Liu and Schutz, 1998; Weiss et al., 2016), whereas others argue that the barrier may be broken in the near future (Boullosa et al., 2011; Joyner et al., 2011; Hoogkamer et al., 2017; Sousa et al., 2018).

With regards to any attempt to break the 2 h barrier, previous research proposed the use of several pacemakers that should be replaced with other athletes as soon as the first ones become fatigued on a loop circuit, as was the case in the Nike Breaking2 attempt (Hoogkamer et al., 2017). However, this strategy is not allowed by the IAAF rules (2015), so the only option is to compete in legitimate races. This is where the Marathon Majors take special importance as since 1998 all world records have been broken in these competitions.

Therefore, the main goal of this study was to analyse the pacing strategies displayed by the winners of the six World Marathon Majors in order to determine which race offers the greatest potential for future world record attempts. We hypothesize that world records are more likely to be achieved in marathons with more even profiles and taking place in stable favorable such as Berlin.

## Methods

Data were gathered from a publicly accessible website (Association of Road Running Statisticians' website, accessed 20 November 2018) providing the winners of the official World Marathon Majors for men between 2006 and 2018, which resulted in a sum of 76 winners. Each set of information included at least: the position, category, official final time, half-marathon time, and time data every 5 km.

The total distance of the marathon was divided into eight sections of 5 km and one section of 2.195 km, and time needed to complete each section was calculated in seconds. Full marathon average speed and the average speed of each section were calculated individually. The relative speed of each section for every runner was then calculated and presented as a percentage of the average speed for the full race.

The athletes were divided into six groups: (A) group of marathon Berlin (winners between 2006 and 2018), (B) group of marathon Boston (winners between 2006 and 2018), (C) group of marathon Chicago (winners between 2006 and 2018), (D) group of marathon London (winners between 2006 and 2018), (E) group of marathon New York (winners between 2006 and 2018, except the marathon of 2012 canceled by a hurricane), (F) group of marathon Tokyo (winners between 2007 and 2018, as before 2007 Tokyo was not part of the Worlds Marathon Majors).

The course information were retrieved through the official internet website for each city marathon, on marathon archive websites and from various media outlets.

### Statistical Analysis

SPSS for Windows version 25.0 (SPSS, Inc., Chicago, IL) was used to analyze the data. Each data set was screened for normality of distribution and homogeneity of variances using a Shapiro-Wilk normality test and a Levene test, respectively. One-way analysis of variance (ANOVA) was used to compare the mean winning time in the last 13 editions of each of the World Marathon Majors. Two-way analysis of variance (ANOVA) with repeated measures for time was used to compare the pacing strategies between the winners of the six world marathons majors. When differences were found, a Tukey's range test was used for *post-hoc* comparisons. The magnitude of the differences or effect sizes (ES) were calculated according to Cohen's d (Cohen, 1998) and interpreted as small (>0.2 and <0.6), moderate (≥0.6 and <1.2), large (≥1.2 and <2.0) or very large (≥2.0 and <4) according to the scale proposed by Hopkins et al. (2009). Significance for all analyses was set at *p* < 0.05.

We acknowledge that such a statistical approach has been criticized as it may induce a greater risk of type I error (Sainani, 2018) and may lead to flawed inference (Sainani et al., 2019). However, it has a practical use in sport science studies (Batterham and Hopkins, 2019).

## Results

**Figure 1:**
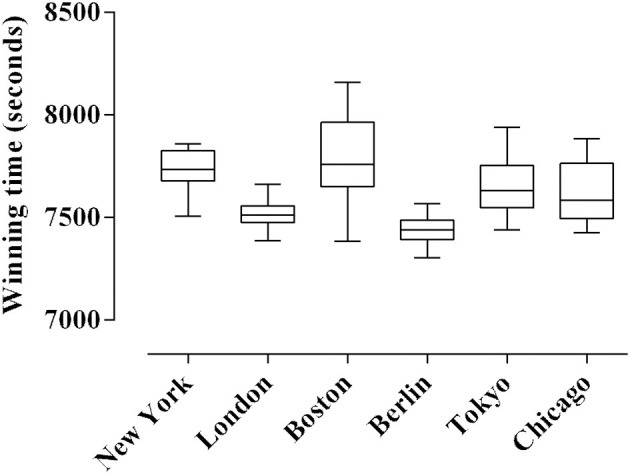
Winning times in the World Marathon Majors in the last 13 years.

Mean winning time in the last 13 editions of each of the World Marathon Majors are presented in Figure 1. We observed differences between New York and London (ES = 1.46, moderate effect, *p* = 0.0030), New York and Berlin (ES = 0.95, small effect, *p* = 0.0001), London and Boston (ES = 0.08, small effect, *p* = 0.0001), Boston and Berlin (ES = 0.10, small effect, *p* = 0.0001), Boston and Chicago (ES = 0.16, small effect, *p* = 0.0361), Berlin and Tokyo (ES = 0.20, small effect, *p* = 0.0034), Berlin and Chicago (ES = 0.27, small effect, *p* = 0.0162).

**Figure 2:**
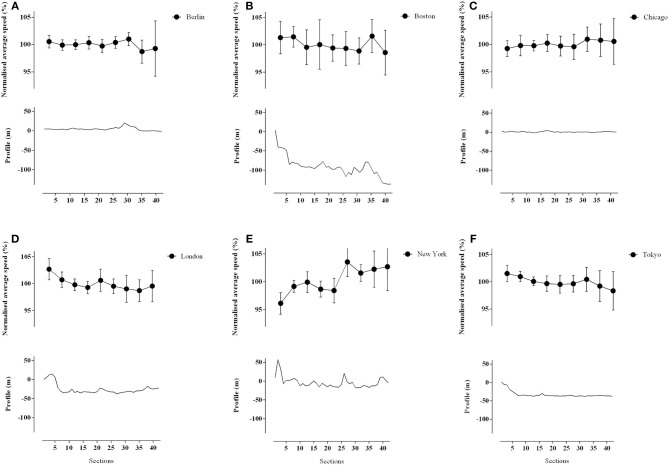
Course profile and normalized average speed of World Marathon Majors' winners by 5 km sections. **(A)** Course profile and normalized average speed of Berlin Marathon. **(B)** Course profile and normalized average speed of Boston Marathon. **(C)** Course profile and normalized average speed of Chicago Marathon. **(D)** Course profile and normalized average speed of London Marathon. **(E)** Course profile and normalized average speed of New York Marathon. **(F)** Course profile and normalized average speed of Tokyo Marathon.

When individual 5 km segments were analyzed, differences were found in running speed relative to the whole race average (Figure 2). In the first 5 km section, New York was slower than Berlin (ES = 2.77, very large effect, *p* = 0.0001), London (ES = 3.36, very large effect, *p* = 0.0001), Tokyo (ES = 3.12, very large effect, *p* = 0.0001), Boston (ES = 2.06, very large effect, *p* = 0.0001), and Chicago (ES = 1.83, large effect, *p* = 0.0133). On the other hand, London was faster than Chicago (ES = 2.00, very large effect, *p* = 0.028).

In section 6, New York again differed from other races, as it was faster than Berlin (ES = 1.55, large effect, *p* = 0.0133), London (ES = 1.89, large effect, *p* = 0.0003), Chicago (ES = 1.60, large effect, *p* = 0.0004), Boston (ES = 1.45, large effect, *p* = 0.0001), and Tokyo (ES = 1.79, large effect, *p* = 0.0008).

In section 8, we found the following differences: Berlin was slower than New York (ES = 0.55, small effect, *p* = 0.0029) and Boston (ES = 1.09, moderate effect, *p* = 0.0257), London was slower than New York (ES = 1.31, large effect, *p* = 0.0027), and Boston (ES = 1.11, moderate effect, *p* = 0.0240) and New York was faster than Tokyo (ES = 0.99, moderate effect, *p* = 0.0211).

In the final 2.2 km section, New York was faster than Berlin (ES = 0.73, moderate effect, *p* = 0.0043), London (ES = 0.85, moderate effect, *p* = 0.0126), Boston (ES = 0.98, moderate effect, *p* = 0.0002), and Tokyo (ES = 1.10, moderate effect, *p* = 0.0001).

## Discussion

Analysis of winning times over a 13 year period indicates clear differences between the six World Marathon Majors (Figure 1). Although there are probably numerous reasons for this, course topography is likely important, especially in terms of its influence on the pacing behaviors displayed. For example, the New York race, which is characterized by substantial undulations, differs from the other races in the initial and final individual 5 km sections. Similarly, New York marathon is the only one of the Majors that consistently allows for a second half marathon faster than the first (ES = 4.90, very large effect). In contrast Berlin, which has the fastest average winning time and is the sight of the current world best performance is relatively flat, starts at an elevation of 38 m above sea level and never exceeds 53 m, and has a net downhill profile over the final 15 km.

The Boston marathon is notable in that we found larger variability in the overall winning times than was the case for the other races. This is in line with a larger analysis performed by Maffetone et al. (2017) who found that the Boston Marathon is characterized by large variability in performances due to external factors such as weather and specifically, the wind direction.

In relation to climate, other aspect that may influence the performance of athletes is temperature (El Helou et al., 2012). It is known that warm weather causes a major alteration of cardiovascular, metabolic, neuromuscular, and thermoregulatory function (Maughan et al., 2007). Consequently, it increases the risk of hyperthermia, directly affecting the central nervous system and contributing to the onset of fatigue during prolonged exercise (Marc et al., 2014). In this sense, El Helou et al. (2012) observed that, the higher the temperature increase, there is a drastic reduction in the running speed and significantly increases the percentage of athletes who retire. For example, in 2007 in the marathon in Chicago, 30.74% of the athletes withdrew, although the organizers tried to interrupt the competition with serious problems of dehydration and thermal shock syndromes (Roberts, 2010). Although we are unable to access data relating to climatic conditions at the time of every individual race, we speculate that variation between locations may at least partially account for the differences in performance we found. For example, with regards to the influence of temperature, Ely et al. (2007) indicated that the best historical times in the marathon have been achieved during the early morning, with cold ambient temperatures (10–15°C) and during the spring or autumn. In this regard, despite the high standard of competition during the summer in the Olympic Games, Continental or International Championships no world records and few good annual performances are established possibly by the temperatures during this period of year (i.e., London Olympics 2012: 27°C, Rio Olympics 2016: 21°C, Doha World Championship 2019: 32°C) (Marc et al., 2014).

One of the most important factors influencing marathon performance is the pacing adopted by athletes (Abbiss and Laursen, 2008; Díaz et al., 2018, 2019). Athletes aim to efficiently use and distribute their energetic resources during athletic competition (Foster et al., 1994), with the aim of using all available reserves before reaching the finish, thereby avoiding premature fatigue (Skorski and Abbiss, 2017) and a significant deceleration before the end (Foster et al., 2004; Hettinga et al., 2007; Tucker and Noakes, 2009). We can observe that in the Tokyo and Boston races, race winners typically slow with increasing distance, and therefore, display positive pacing profiles, unlike Berlin where the athletes display a more uniform speed throughout the whole race (Figure 2). Interestingly, the New York profile favors a fast end that never compensates for the slow pace of the first half, which reinforces the importance of a stable and constant intensity to perform optimally. There is evidence to suggest that, in a marathon, a pacing strategy characterized by very little speed changes across the race is optimal if the goal is to run as fast as possible (Angus, 2014). This observation is in line with that made by Díaz et al. (2018) who assessed the historical development of pacing strategies in world best marathon performances. This ability to achieve such a uniform pacing strategy may be enhanced through the use of designated “pacemakers” who may reduce the psychological burden of regulating speed in competition, and thereby improve performance (Zouhal et al., 2015). During a 3000 m running time trial, presence of a pacemaker resulted in lower blood lactate concentrations and reduced RPE (Zouhal et al., 2015). Running with pacemakers also helps eliminate air resistance, thereby saving energy and reducing oxygen consumption by 8% at a speed of 21.5 kph (Pugh, 1970). Furthermore, Rauch et al. (2013) suggested that pacemakers can act as a placebo, increasing the motivation of the athletes to maintain or increase the pace in the final kilometers.

Finally, it is important to emphasize that organizational and traditional factors specific to the individual races may help explain some of the findings of this analysis. For example, the Boston race, and since 2007, the New York race, do not use pacemakers to assist athletes in achieving faster times. In explaining the reasons for this, organizers of the New York race say the presence of pacemakers makes the competitions lose their essence because the athletes do not “start running” until the pacemakers drop out (Mehaffey, 2009). Furthermore, the performances of elite runners may be influenced by the prize money (Maffetone et al., 2017). The winners of the World Marathon Majors receive a prize of $250,000 in addition to an event specific prize that differs between races as well as bonuses for breaking world records. Times set at Boston are ineligible for record purposes due to the distance of the start line from the finish line which could result in favorable prevailing winds. Given that economic reasons are a primary motivational factor for East African runners (Onywera et al., 2006), it would therefore seem unlikely that an athlete considered to have a realistic chance of achieving a world best performance would attempt to do so at Boston, regardless of the course profile.

## Conclusion

We have identified differences in overall performance and pacing behavior displayed by winners of the mens races over the last 13 editions of the World Marathon Majors series. Although these differences may be partially accounted for by course topography and environmental conditions, it seems likely that organizational issues and traditions may also have an influence. In terms of potential venues for future world record attempts, then historical data suggests that Berlin, which owns the 7 best performances of all time over this distance in men, is the most likely candidate whilst such a performance is unlikely in New York or Boston (which is ineligible anyway).

## Data Availability Statement

All datasets generated for this study are included in the article/supplementary material.

## Author Contributions

JD participated in the study design, collected all the data and participated in data analysis, statistical analysis, interpretation of the data, and manuscript editing. AR participated in the study design, participated in data analysis, statistical analysis, interpretation of the data, and manuscript editing. EF-O participated in the conception and design of the study and revised/reviewed the manuscript. MT designed and carried out these experiments, participated in data analysis, and statistical analysis. JS-C participated in the coordination of our study participated in data analysis, statistical analysis, interpretation of the data, and helped to polish the manuscript. All authors have read and approved the final version of the manuscript and agreed with the order of presentation of the authors.

### Conflict of Interest

The authors declare that the research was conducted in the absence of any commercial or financial relationships that could be construed as a potential conflict of interest.
